# Association between ideal cardiovascular health and the atherogenic index of plasma

**DOI:** 10.1097/MD.0000000000003866

**Published:** 2016-06-17

**Authors:** Shiwei Shen, Yun Lu, Huajin Qi, Feng Li, Zhenhai Shen, Liuxin Wu, Chengjian Yang, Ling Wang, Kedong Shui, Yaping Wang, Dongchang Qiang, Jingting Yun, Xiaofeng Weng

**Affiliations:** a Wuxi No.2 People's Hospital Affiliated to Nanjing Medical University, Wuxi, Jiangsu, China; b The Taihu Rehabilitation Hospital of Jiangsu Province, Wuxi, Jiangsu, China; c Jiangsu Provincial Research Center for Health Assessment and Intervention, Wuxi, Jiangsu, China; d Health Management Branch of Chinese Medical Association, Beijing, China.

**Keywords:** atherogenic index of plasma, atherosclerosis, ideal cardiovascular health

## Abstract

The American Heart Association aims to improve cardiovascular health by encouraging the general population to meet 7 cardiovascular health behaviors and factors. The atherogenic index of plasma (AIP) is an important index. Our aim is to evaluate the relationship between ideal cardiovascular health and the atherogenic index of plasma (AIP) in middle-aged Chinese men.

A cross-sectional study was performed. A total of 27,824 middle-aged Chinese men were enrolled. The association between ideal cardiovascular health behaviors and factors and AIP was determined. The 7 cardiovascular health metrics were scored as follows: 0, poor; 1, general; and 2, ideal. The cardiovascular health status was classified according to the total score, as follows: 0 to 4, inadequate; 5 to 9, average; and 10 to 14, optimum. Analyses assessed the prevalence of 7 cardiovascular health metrics, its association with AIP. Logistic regression models were used to calculate odds ratios (ORs), adjusting for age.

All 7 cardiovascular health metrics were shown to correlate with AIP (all *P* values < 0.05), and the strongest correlation existed between body mass and AIP, followed by total cholesterol and AIP. The mean AIP level increased with the decrease in the score of each of the 7 cardiovascular health metrics (all *P* values < 0.05). The subjects with poor cardiovascular health status had a 4.982-fold increase in the high risk of developing atherosclerosis, whereas a 1-point increase in the cardiovascular health score resulted a 0.046 reduction in AIP and a 22.3% reduction in the high-risk of developing atherosclerosis (*OR* = 0.777, 95% CI: 0.768–0.787).

The ideal cardiovascular health score correlated significantly with AIP, and a 1-point increase in the cardiovascular health score led to a 0.046 reduction in AIP and a 22.3% reduction in the high risk of developing atherosclerosis. These validated the value of ideal cardiovascular health behaviors and factors in the prediction of high risk of developing cardiovascular diseases. Ideal cardiovascular health metrics are of great realistic significance for the prevention and control of atherosclerosis and cardiovascular diseases.

## 1 INTRODUCTION

Cardiovascular disease has become a global public health concern.^[1]^ The 2013 Report on Cardiovascular Diseases in China estimated that ∼290 million people have cardiovascular diseases in China.^[2]^ In contrast, the number of deaths due to cardiovascular diseases has shown a decline in the United States during the recent 20 years, 50% of which is attributed to a reduction in blood pressure and the blood cholesterol level, and smoking cessation.^[3]^ Ideal cardiovascular health, which was proposed by the American Heart Association (AHA) in 2010, has been shown to be protective against cardiovascular and cerebrovascular diseases.^[4–8]^ The atherogenic index of plasma (AIP), the logarithm of the molar ratio of triglyceridemia to high-density lipoprotein cholesterol (TG/HDL-C), has shown a strong association with the diameter of low-density lipoprotein cholesterol (LDL-C) particles.^[9]^ With an elevation in the AIP, the proportion of small, dense LDL (sdLDL) increases.^[10–12]^ sdLDL is a subtype of LDL subfraction with high density and small size, and may cause atherosclerosis. In 2002, the National Cholesterol Education Program (NCEP) included sdLDL as a new risk factor of coronary heart disease, and recommended measurement of sdLDL.^[13]^ The present study was designed to determine the association between ideal cardiovascular health behaviors and factors and the AIP among middle-aged men in southeastern China to provide data for the development of preventive and control strategies for cardiovascular diseases.

## 2 SUBJECTS AND METHODS

### 2.1 Subjects

A cross-sectional study was performed. The men between 40 and 64 years of age receiving health examinations in our hospital from 1 January 2014 through 30 June 2015 were recruited, and all recruited subjects resided in the Suzhou, Wuxi, and Changzhou regions of southeastern China. The study exclusion criteria included the following: use of lipid-regulating drugs; a history of myocardial infarction or stroke; severe hepatic or renal insufficiency; or incomplete medical records. A total of 27,824 patients met the appropriate criteria.

The study protocol was approved by the Ethics Review Committee of our hospital. Written informed consent was obtained from all participants following a detailed description of the purpose of this study.

### 2.2 Questionnaire survey

Demographic and clinical characteristics were captured using a self-designed questionnaire, including age, residency, profession, smoking status, alcohol consumption, salt consumption, living habits, physical activity status, medical history of chronic diseases (hypertension, diabetes, coronary heart disease, stroke, and other cardiovascular diseases), and medications. The questionnaire was administered by well-trained medical professionals.

### 2.3 Measurement of cardiovascular risk factors

All subjects had measurements of height, weight, waist circumstance (WC), systolic blood pressure (SBP), diastolic blood pressure (DBP), and body mass index (BMI). In addition, all participants fasted for 8 to 12 hours, and 5 mL of venous blood was collected from the cubital vein the following morning. The serum levels of TG, total cholesterol (TC), HDL-C, and LDL-C were determined using the glycerol phosphate oxidase method, the oxidase method, an antibody-based homogeneous assay, and the homogeneous assay on a fully automatically biochemical analyzer (Hitachi 7600; Hitachi, Ltd., Tokyo, Japan), respectively.

### 2.4 Grouping of atherosclerosis risk

The AIP was calculated using the following formula: AIP = log (TG/HDL-C). All participants were assigned to 1 of 2 groups based on the AIP. Subjects with an AIP ≤ 0.21 were assigned to the low- or moderate-risk atherosclerosis group, whereas subjects with an AIP > 0.21 were assigned to the high-risk atherosclerosis group.^[14]^

### 2.5 Definition of cardiovascular health

Based on the definition of cardiovascular health proposed by the AHA in 2010,^[15]^ vegetable intakes were changed to salt intake in this study. Physical activity was defined as moderate-intensity aerobic exercise, including fast walking, running, bicycle riding, rope skipping, and swimming and the classification criterion of physical activity was adjusted.

In accordance with AHA definitions, 7 Cardiovascular Health metrics were classified into ideal, intermediate, and poor: (1) smoking: ideal (never or quit > 1 year), intermediate (quit < 1 year), and poor (current); (2) body mass index (BMI): ideal (<25 kg/m^2^), intermediate (25 to < 30 kg/m^2^), and poor (≥30 kg/m^2^); (3) physical activity: ideal (physical activity ≥ 3 times a week, with > 30 minutes each time or physical activity > 90 minutes per week), intermediate (physical activity of < 3 times a week, with < 30 minutes each time or ≤ 89 minutes of physical activity per week), and poor (no extra physical activity except daily life and work activities); (4) salt intake: ideal(< 6 g/d), intermediate (6–12 g/d), and poor (>12 g/d) based on responses to questions related to salt preferences; (5) total cholesterol (TC): ideal (untreated and < 5.2 mmol/L [200 mg/dL]), intermediate (treated to < 5.2 mmol/L or 5.2–6.2 mmol/L), and poor (>6.2 mmol/L [240 mg/dL]); (6) blood pressure (BP): ideal (untreated and < 120/ < 80 mm Hg), intermediate (treated to < 120/ < 80 mm Hg or 120–139/80–89 mm Hg), and poor (≥140/90 mm Hg); and (7) fasting plasma glucose (FPG): ideal (untreated and < 5.6 mmol/L [100 mg/dL]), intermediate (treated to < 5.6 mmol/L or 5.6–7.0 mmol/L), and poor (≥7.0 mmol/L [125 mg/dL]).

For each subject, the 7 cardiovascular health metrics were scored as follows: 0, poor; 1, general; and 2, ideal. The sum of the scores of the 7 cardiovascular health metrics was defined as the total cardiovascular health score, and cardiovascular health status was classified according to the total score, as follows: 0 to 4, inadequate; 5 to 9, average; and 10 to 14, optimum.^[16]^

### 2.6 Statistical analysis

The AIP were described as the mean ± standard deviation (SD), whereas the distribution of ideal cardiovascular health components and number of ideal cardiovascular health metrics were expressed as a number (proportion). AIP and atherosclerosis risk were calculated and compared according to categories of cardiovascular health behaviors and factors using analysis of variance (ANOVA) and χ^2^ statistics, respectively. For each ideal cardiovascular health component, atherosclerosis risks were calculated. Logistic regression models were used, adjusting for age. Odds ratio (OR) of atherosclerosis risk across ideal cardiovascular health component categories (intermediate or poor versus ideal) and overall cardiovascular health categories (inadequate or average versus optimum) were calculated. Ideal cardiovascular health score was also examined as a continuous variable, considering the OR per a 1-point higher overall ideal cardiovascular health score. All statistical analyses were conducted using SPSS version 16.0 (SPSS, Inc., Chicago, IL), with a 2-tailed *P* value < 0.05 considered statistically significant.

## 3 RESULTS

### 3.1 Baseline cardiovascular health metrics and AIP

A total of 27,824 subjects were enrolled in this study, and the subjects at 40 to 49, 50 to 59, and 60 to 64 years of age consisted of 45.4%, 41.2%, and 13.4% of all study subjects, respectively. The percentages of the ideal health metrics were as follows: smoking, 40.3%; BMI, 50.2%; physical activity, 45.6%; salt intake, 15.6%; TC, 68.5%; blood pressure, 22.5%; and fasting blood glucose (FBG), 66.9% (see Table 1). The mean AIP level was shown to increase with a decrease in the score of each of the 7 cardiovascular health metrics (all *P* values < 0.05). A comparable AIP level was observed between the nonexercise and occasional exercise groups; however, a significant decline of AIP was noted in the frequent exercise group (*P* < 0.05).

**Table 1:**
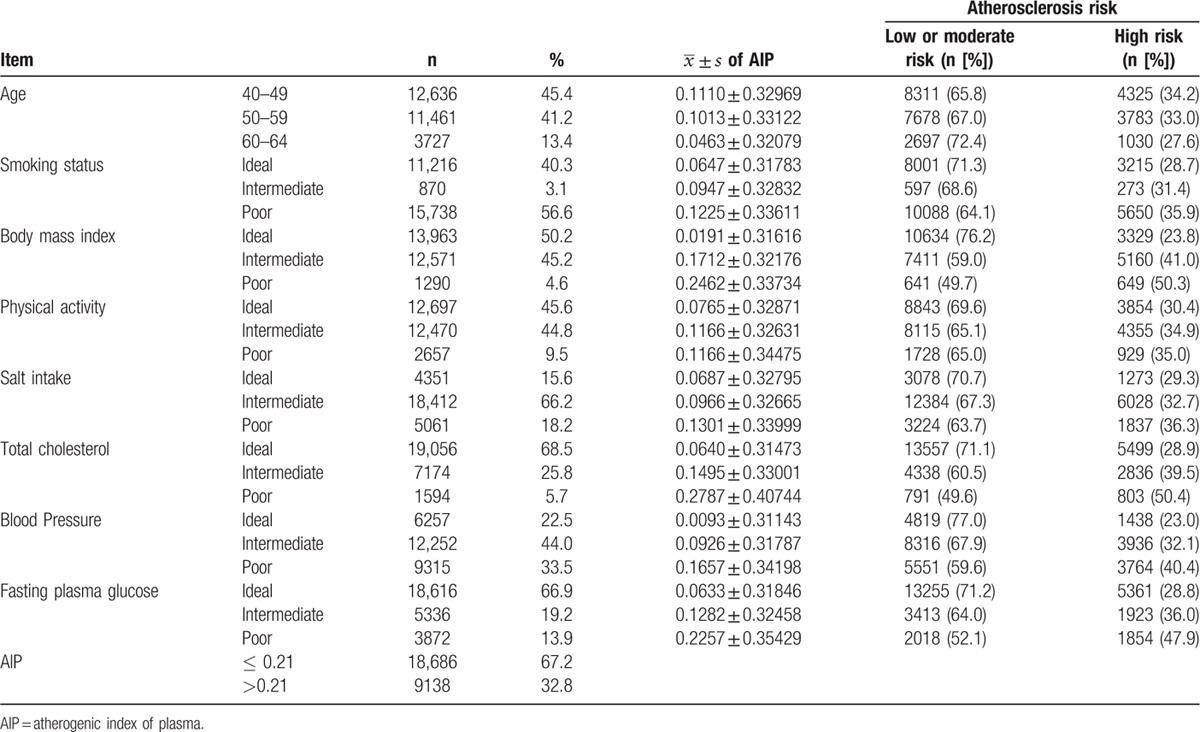
Baseline cardiovascular health metrics and AIP.

### 3.2 Correlation between 7 cardiovascular health metrics and AIP

AIP grouping served as a dependent variable, and the 7 cardiovascular health metrics were included in the logistic regression model as independent variables with age adjustment, logistic regression analysis revealed that smoking status, BMI, physical activity, salt intake, TC, blood pressure, and FBG had a remarkable effect on AIP (all *P*-values < 0.05), with the strongest correlation between BMI and TC and AIP (see Table 2).

**Table 2:**
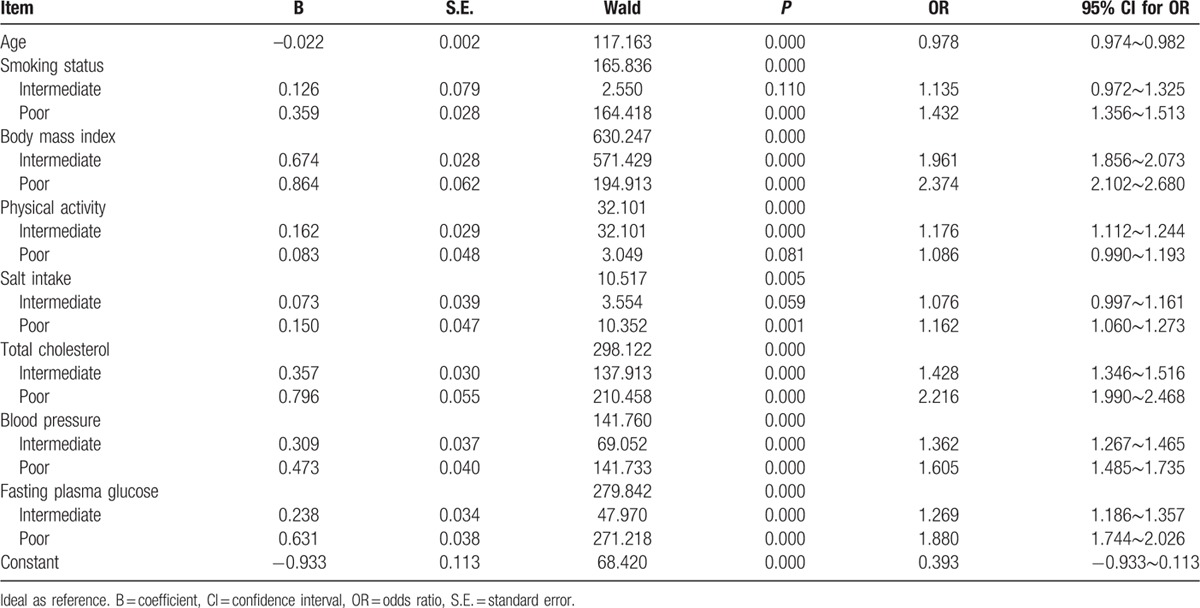
Seven ideal cardiovascular health metrics and atherosclerosis risk.

### 3.3 Correlation between cardiovascular health status and AIP

**Figure 1:**
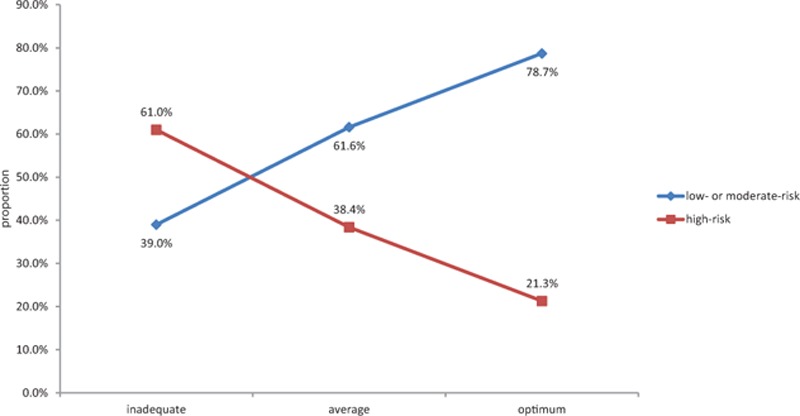
Atherosclerosis risk at different cardiovascular health status.

There were 61.0%, 38.4%, and 21.3% of the subjects with inadequate, average, and optimum cardiovascular health status at high risk for atherosclerosis (χ^2^ = 1178.878, *P* = 0.000) (see Fig. 1). Logistic regression analysis showed that the subjects with average cardiovascular health had a 1.350-fold increase in the risk of atherosclerosis compared to those with optimum cardiovascular health after adjusting for age (*OR* = 2.350, 95% CI: 2.220–2.488), whereas the risk of atherosclerosis increased by 4.982-fold in the patients with poor cardiovascular health relative to those with optimum cardiovascular health (*OR* = 5.982, 95% CI: 5.191–6.894) (see Table 3).

**Table 3:**
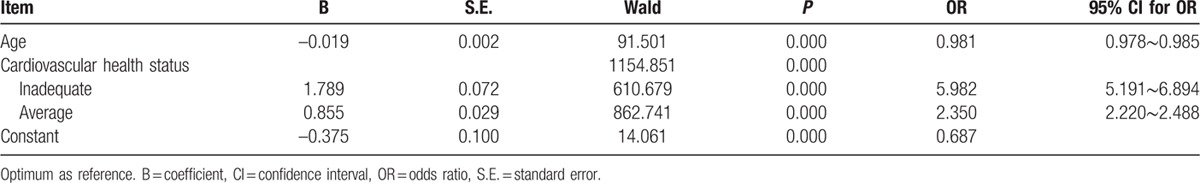
Cardiovascular health status and atherosclerosis risk.

### 3.4 Relationship between cardiovascular health score and AIP

**Figure 2:**
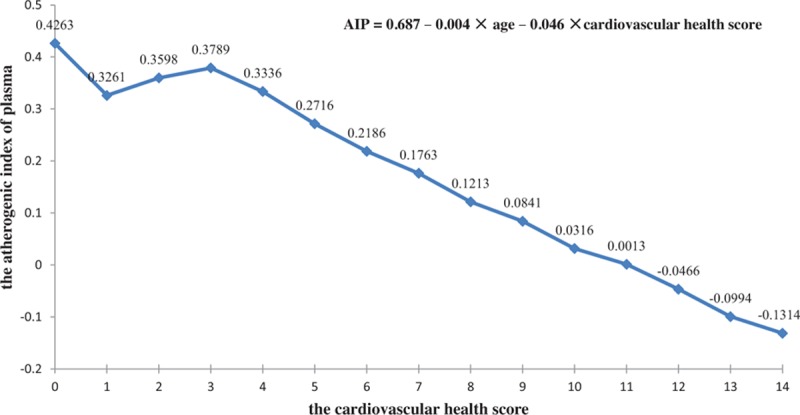
Relationship between the cardiovascular health score and AIP. AIP = atherogenic index of plasma.

If AIP served as a dependent variable and cardiovascular health score as an independent variable, regression analysis showed that a 1-point increase in the cardiovascular health score led to a 0.046 reduction in the AIP level (see Fig. 2). In addition, logistic regression analysis revealed that after adjusting for age, a 1-point increase in the cardiovascular health score resulted in a 22.3% reduction of high risk of developing atherosclerosis (*OR* = 0.777, 95% CI: 0.768–0.787).

## 4 DISCUSSION

As ideal cardiovascular health was first proposed and defined by the AHA in 2010, the prevalence of ideal cardiovascular health has been reported worldwide; however, the cardiovascular health metrics and scores vary due to differences in country, race, region, economy, and lifestyle.^[4–6,8,17,18]^

In the present study, we found only 133 of 27,824 middle-aged Chinese men exhibited ideal levels of all 7 cardiovascular health metrics, with a prevalence of 0.478%. Only 0.5% of the 9962 urban participants from the survey of the Disease Risk Evaluation and Health Management study from October 2009 to February 2012 were found to meet ideal levels of all 7 cardiovascular health metrics.^[19]^ The 2003–2008 National Health and Nutrition Examination Surveys (NHANES) estimated that <1% of adults exhibited ideal cardiovascular health for all 7 metrics in the United States,^[20]^ whereas the prevalence of meeting all 7 cardiovascular health metrics was 0.67% in middle-aged men in Korea.^[21]^ Our findings were nearly in agreement with previous reports. In addition, our findings showed that the lowest prevalence of salt intake (15.6%) and blood pressure (22.5%) met the ideal levels in the 7 cardiovascular health metrics, which was similar to the previous studies reporting a daily salt intake of >12 g per person in most areas of China, and showing that a high-salt diet is a major risk factor for developing hypertension in China.^[22,23]^ The results of this study validate a low prevalence of ideal cardiovascular health in Chinese adults.

Our findings showed that the score of each of the 7 cardiovascular health metrics and the total score of the 7 health metrics significantly correlated with AIP, and the mean AIP level increased with a decrease in the score of each of the 7 health metrics (all *P*-values < 0.05). Among the 7 cardiovascular health metrics, BMI exhibited the greatest effect on AIP, followed by the TC level, FBG level, smoking status, blood pressure, physical activity, and salt intake. The strongest correlation between BMI and AIP may be attributable to the most overweight and obese subjects having elevated TG. The large impact of TC on AIP is considered to be due to HDL-C as a component of TC. Nevertheless, TG and HDL-C directly affect AIP.

It was noted that the subjects with inadequate cardiovascular health status had a 4.982-fold increase in the risk of atherosclerosis than subjects with optimum cardiovascular health status (*OR* = 5.982, 95% CI: 5.191–6.894). Logistic regression analysis showed that a 1-point increase in the cardiovascular health score led to a 0.046 reduction in AIP, and a 1-point increase in the cardiovascular health score, resulted in a 22.3% reduction of high risk of developing atherosclerosis after adjusting for age. The results from the Reasons for Geographic and Racial Differences in Stroke (REGARDS) showed a clear-cut reduction in the risk of incident stroke in subjects with average or optimum cardiovascular health status than those with inadequate cardiovascular health status, and that each better health category of cardiovascular health status was associated with a 25% lower risk of stroke (hazard ratio, 0.75; 95% CI: 0.63–0.90), and a 1-point higher cardiovascular health score was associated with an 8% lower risk of stroke (hazard ratio, 0.92; 95% CI: 0.88–0.95).^[16]^ These findings indicate that the subjects with inadequate cardiovascular health are at high risk for atherosclerosis and cardiovascular diseases. Thus, health education and promotion and lifestyle interventions tailored to these high-risk populations are considered to improve the cardiovascular health behaviors and factors, which will undoubtedly result in a reduction in the prevalence of atherosclerosis and cardiovascular diseases.

Currently, China is at a stage of shifting to a Western diet and urbanization lifestyle, and the overall status or changing trend of the cardiovascular health behaviors and factors is not optimistic.^[5,17,19,24]^ The concept of ideal cardiovascular health behaviors and factors, and their value in predicting the high risk of developing cardiovascular diseases should be popularized in community populations to promote the health among high-risk populations with poor cardiovascular health. Notably, poor lifestyles should be improved among middle-aged populations with smoking, high working pressure, inadequate physical activity, and overweight/obesity based on the 7 cardiovascular health metrics. First, smoking cessation and tobacco control should be implemented. Second, adipose and salt intake should be reduced in the diet structure. Third, the duration of exercise should be extended, such as > 90 minutes of moderate-intensity aerobic exercise a week. Finally, the body weight should be strictly controlled. It is reported that the Asian population has a significantly higher body fat percentage than the Western population with the same BMI.^[25]^ In addition, the blood pressure, and blood lipid and glucose levels should be monitored periodically and timely adjustment of medications is suggested.

The present study had the following limitations: (1) vegetable and fruit intake was replaced by salt intake; (2) ideal physical activity was defined as > 90 minutes of moderate-intensity aerobic exercise a week, which is different from the criteria proposed by the AHA; and (3) the study subjects were middle-aged men. Further studies to determine the correlation between cardiovascular health and the AIP are warranted.

## 5 CONCLUSIONS

The cardiovascular health score correlated significantly with AIP, and a 1-point increase in the cardiovascular health score led to a 0.046 reduction in AIP and a 22.3% reduction in the high risk of developing atherosclerosis. AIP validates the value of ideal cardiovascular health behaviors and factors in the prediction of high risk of developing cardiovascular diseases. Ideal cardiovascular health metrics are of great realistic significance for the prevention and control of atherosclerosis and cardiovascular diseases.
